# Morphological and ITS-Based Molecular Characterization of Dermatophytes from Pets and In Vitro Antifungal Evaluation of Muğla Propolis

**DOI:** 10.3390/vetsci13020136

**Published:** 2026-01-29

**Authors:** Yalçın Semiha, Yüksek Rumeysa, Özgen Arzu, Sorucu Ali, Cengiz Seyda

**Affiliations:** 1 Department of Microbiology, Faculty of Milas Veterinary Medicine, Muğla Sıtkı Koçman University, Muğla 48200, Türkiye; 2 Department of Microbiology, Faculty of Veterinary Medicine, Aydın Adnan Menderes University, Aydın 09020, Türkiye; 3 Department of Medical Laboratory Techniques, Vocational School of Health Services, İstanbul Gelişim University, Istanbul 34310, Türkiye; 4 Department of Pharmacology, Faculty of Milas Veterinary Medicine, Muğla Sıtkı Koçman University, Muğla 48200, Türkiye

**Keywords:** cat, dermatophytes, dog, *Microsporum canis*, *Nannizea gypsea*, propolis

## Abstract

Dermatophytosis is a common zoonotic fungal infection in companion animals, most frequently caused by *Microsporum canis*, while the geophilic species *Nannizzia gypsea* may occasionally infect cats. Conventional morphological identification of dermatophytes is often challenging due to phenotypic similarities, underscoring the importance of molecular confirmation. In this study, dermatophyte field isolates obtained from cats with suspected dermatophytosis were identified using cultural characteristics and Internal Transcribed Spacer (ITS) region sequencing. Phylogenetic analysis based on ITS sequences showed that the isolates were highly similar to each other and clustered closely with reference strains and previously reported dermatophyte strains from different geographical regions. Subsequently, the in vitro antifungal activity of a propolis extract collected from the Muğla region (Türkiye) was evaluated using the agar dilution method at concentrations ranging from 6.25 to 100 mg/mL. At all tested concentrations, propolis inhibited mycelial growth in all four molecularly confirmed dermatophyte field isolates, whereas substantial growth was observed in the negative control plates. These findings indicate that Muğla propolis exhibits in vitro antifungal activity at the tested concentrations against dermatophyte field isolates and warrants further investigation as a potential natural antifungal source.

## Simple Summary

Dermatophytosis is a fungal skin disease that commonly affects pets and can be transmitted to humans. Accurate identification of the causative dermatophytes is essential for appropriate diagnosis and control. In this study, dermatophyte isolates obtained from cats with suspected dermatophytosis were identified using conventional mycological methods and ITS-based molecular analysis. Phylogenetic analysis showed that the isolates were closely related to each other and clustered with dermatophyte strains previously reported from different geographical regions and reference databases. In addition, the in vitro antifungal activity of Muğla-origin propolis was evaluated. Propolis completely inhibited the growth of all tested dermatophyte field isolates at the tested concentrations. These findings suggest that Muğla propolis may represent a promising natural candidate for further investigation against dermatophyte infections.

## 1. Introduction

Dermatophytes are filamentous fungi that can infect keratinized tissues in both animals and humans, with infections particularly caused by zoonotic species such as *Microsporum canis*, *Trichophyton mentagrophytes*, and *Trichophyton verrucosum* [1]. These agents exhibit tropism for keratinized structures and lead to superficial dermatological infections. Dermatophytosis in cats and dogs is characterized by clinical signs such as erythema, scaling, broken hairs, and focal or multifocal alopecia, which are among the most frequently reported manifestations of the disease [2,3]. Due to the typically circular configuration of these superficial lesions, the infection is commonly referred to as “ringworm” in both humans and animals [4,5,6]. Epidemiological studies conducted in different geographical regions, including Europe, South America, North America, and Türkiye, have reported that the prevalence of dermatophytosis ranges from approximately 4% to 20% in dogs and exceeds 20% in cats [7,8,9,10,11].

Dermatophytes are classified as anthropophilic, zoophilic, and geophilic species according to their ecological niches [12]. Although numerous dermatophyte species have been isolated from domestic animals, the majority of clinical cases are associated with the zoophilic *species M. canis*, *T. mentagrophytes*, *Trichophyton quinckeanum*, and *T. verrucosum*, as well as the geophilic species *M. gypseum* [11]. *M. canis*, a member of the genus *Microsporum*, is a typical zoophilic dermatophyte. It produces macroconidia that are typically spindle-shaped, rough-walled, and thick-walled, with thinner septa, measuring approximately 35–110 × 12–25 µm. These macroconidia are 6–12-celled, verrucose, and often exhibit a slightly curved body with a rostrate apex. Another relevant dermatophyte species, *Nannizzia gypsea* (formerly classified as *Microsporum gypseum*), is a geophilic dermatophyte with soil as its natural reservoir and may cause opportunistic infections in animals. It forms macroconidia in large clusters, which are fusiform, regularly verrucose, and relatively thin-walled, measuring approximately 25–60 × 8.5–15 µm, and consisting of 3–6 (occasionally up to 8) cells [13,14]. Cats are considered the natural hosts and carriers of *M. canis*. Not all cats exposed to spores develop infection; some animals may remain clinically healthy while mechanically carrying spores on their hair coat and are therefore defined as asymptomatic carriers [4]. The clinical presentation of *M. canis* infection is highly variable, and the absence of pathognomonic clinical signs may lead to confusion with other dermatological conditions [3]. *N. gypsea* has been reported as an occasional cause of dermatophytosis in domestic animals, particularly in cases associated with environmental exposure, but is encountered far less frequently than *M. canis* in routine veterinary practice [2,3,4].

Laboratory diagnosis of dermatophytes begins with direct microscopic examination of clinical samples to demonstrate hyphal and arthroconidial structures, followed by species-level identification through evaluation of colony morphology (growth rate, topography, pigmentation), microscopic conidial structures, and various physiological characteristics [13,14,15]. However, similarities in phenotypic characteristics, pleomorphism, and variations related to culture conditions can make identification based solely on morphology challenging [16]. Wood’s lamp examination may be useful as an auxiliary screening method, particularly in *M. canis* infections, but it is not diagnostic on its own and requires confirmation [3]. For this reason, fungal culture is considered the fundamental and reliable diagnostic method for confirming dermatophytosis [2,3]. In recent years, molecular methods such as PCR and sequencing of the ITS region have been increasingly used as complementary diagnostic tools to confirm species-level identification of dermatophytes isolated in culture, particularly in cases involving mixed infections [17].

Propolis is a complex bee product formed when honeybees (*Apis mellifera*) mix plant-derived resins, mucilage, gum, latex, and pollen with their enzymes and beeswax [18]. Bees use propolis for various purposes, such as repairing the hive, disinfecting it, and protecting it against external factors. The antimicrobial effect of propolis arises from its rich content of phenolic compounds, which protect the hive from bacterial, fungal, and viral infections [18,19]. In general, propolis consists of 50% resins, 30% beeswax, 10% volatile and aromatic oils, 5% pollen, and 5% other compounds [19]. The phenolic compounds within the resin fraction are highly diverse, with flavonoids, phenolic acids, and their derivatives forming the predominant groups [20].

While phenolic compounds in plants play various biological roles such as protection against pathogens and UV radiation, and regulation of metabolic processes, contributing to color and flavor formation, flavonoids have been termed functional foods or bioflavonoids due to their numerous biological effects in the field of health [21,22,23]. In humans and animals, these compounds exhibit a wide range of biological activities, including antibacterial, antifungal, antiviral, antioxidant, anti-inflammatory, immunomodulatory, and antitumoral effects [24,25,26]. Among these activities, the antifungal potential of propolis has attracted particular attention, and several studies have reported its activity against yeasts, dermatophytes, and non-dermatophytic molds; however, when compared with the extensive literature on *Candida* spp. and filamentous fungi, studies specifically focusing on dermatophytes, particularly molecularly confirmed field isolates, remain limited [27,28,29,30,31,32]. In this study, *Microsporum* spp. field isolates were obtained from skin scrapings collected from cats and dogs with suspected dermatophytosis and were identified using both morphological and ITS-based molecular methods. The in vitro antifungal activity of a propolis extract from the Muğla region was evaluated against molecularly confirmed field isolates, and the extract’s phenolic composition was determined. Considering the growing concern over antifungal resistance and the consequent restrictions of existing treatment options [33], this study intends to offer preliminary data on the antifungal efficacy of Muğla propolis against dermatophytes.

## 2. Materials and Methods

### 2.1. Sampling, Isolation, and Morphological Identification of Dermatophyte Agents

This study was approved by the Local Ethics Committee for Animal Experiments of Muğla Sıtkı Koçman University, Directorate of the Experimental Animals Application and Research Center (Approval No. 2023/01; Date: 6 January 2023). Animals were included in this study based on the presence of clinical skin lesions compatible with dermatophytosis and a clinical suspicion of fungal infection. All samples were collected from cats and dogs presented to veterinary clinics in the Muğla region, Türkiye, as part of routine clinical examinations. No additional inclusion or exclusion criteria regarding age, sex, breed, housing conditions, or prior antifungal treatment were applied, as this study was designed to reflect routine clinical cases encountered in veterinary practice. Accordingly, skin scrapings and hair samples were collected from a total of thirty cats and dogs suspected of dermatophytosis.

The affected areas were first treated with 70% alcohol, and once the alcohol dried, the scrapings were taken from the outermost part of the lesion by gently scraping with a sterile scalpel and placed in sterile containers. Hair was also plucked from the same area along with the skin scrapings. The samples brought to the laboratory underwent further processing, where hair and skin pieces were embedded in Sabouraud Dextrose Agar (SDA) using a sterile loop and scalpel and cultured. The media were incubated at 25 °C for 1–4 weeks and monitored daily. The growth status and duration of the colonies formed after incubation were evaluated macroscopically by examining the colony color on both the front and back sides of the Petri dish and the types of colonies formed. Microscopic examinations were conducted to identify fungal agents from the colonies. For microscopic evaluation, a portion of the reproduced colonies was taken with a loop and examined on a clean slide with lactophenol cotton blue. The structures of the hyphae, macroconidia, and microconidia of the colonies were assessed, and the agents were identified at the genus level. The fungi identified as dermatophyte agents were transferred into SDA media and stored at +4 °C until used for molecular analysis [34].

### 2.2. Molecular Identification and Characterization of Dermatophyte Agents

#### 2.2.1. DNA Extraction, Amplification, and Sequence Analysis of Dermatophytes

DNA extraction, PCR amplification, and Sanger sequencing were outsourced to BM Labosis (Ankara, Türkiye). Genomic DNA was extracted from fungal isolates using the GeneMATRIX Plant & Fungi DNA Isolation Kit (EURx Sp. z o.o.; Gdansk, Poland) according to the manufacturer’s instructions. The concentration and purity of the extracted DNA were assessed using a NanoDrop spectrophotometer (Thermo Scientific NanoDrop 2000, Thermo Fisher Scientific, Waltham, MA, USA) by measuring absorbance ratios at 260/280 nm. PCR products were subjected to Sanger sequencing, and sequence quality was evaluated by visual inspection of chromatograms. Low-quality sequence regions were trimmed prior to downstream analysis.

To determine the species of the fungal isolates, internal transcribed spacer (ITS) DNA regions were amplified by polymerase chain reaction (PCR) using ITS1 (5′ TCCGTAGGTGAACCTGCGG 3′)–ITS4 (5′ TCCTCCGCTTATTGATATGC 3′) universal primers [35,36]. The PCR reaction (35 µL) consisted of the following components: 0.2 mM dNTP mix, 0.3 µM of each primer, 10 ng/µL DNA, 1X PCR Buffer, 1.5 mM MgCl2, and 2 U/µL of Taq polymerase (FIREPol^®^ DNA Polymerase, Solis BioDyne OÜ, Tartu, Estonia). The reaction was carried out in a Kyratec Thermal Cycler with the following PCR conditions: an initial denaturation and enzyme activation step at 95 °C for 5 min, followed by 40 cycles of amplification. Each cycle included denaturation at 95 °C for 45 s, annealing at 57 °C for 45 s, and extension at 72 °C for 60 s. The protocol concluded with a final extension at 72 °C for 5 min. The PCR products were separated using 1.5% agarose gel electrophoresis at 100 V for 90 min and subsequently visualized. The ITS DNA region fragment, approximately 700 bp in length, was excised and purified using the PCR Clean-up System (MAGBIO HighPrep™ PCR Clean-up System, MAGBIO Genomics, Inc., Gaithersburg, MD, USA). The concentration of the purified ITS DNA amplicon was then measured using a Nanodrop. Samples prepared for Sanger sequencing were sequenced at the Macrogen Netherlands laboratory using an ABI 3730XL Sanger sequencer (Applied Biosystems, Foster City, CA, USA) along with the BigDye Terminator v3.1 Cycle Sequencing Kit (Applied Biosystems, Foster City, CA, USA).

#### 2.2.2. Identification of Dermatophytes Using Gene Sequences

The authors conducted the processes of contig assembly, sequence quality evaluation, and species identification. Raw sequence reads obtained with ITS1 and ITS4 primers were assembled into contigs using the Contig Assembly Program (CAP) algorithm implemented in BioEdit software (version 7.0.5) to generate consensus sequences. The resulting nucleotide sequence data was then searched in GenBank. The obtained ITS sequences, approximately 678–789 bp in length, were compared with the sequences available in NCBI GenBank to determine their similarity rates and confirm the species assignment. The ITS DNA sequences of fungal isolates were registered in the GenBank, and the accession numbers were acquired. For phylogenetic analysis, the ITS sequences of 33 species closely related to the fungal isolates in our study (GenBank similarity rates: 99.73–100%) were used. ITS DNA sequences of other fungal isolates from animals (horse, dog, cat, equids, cow, hamster, and rodent), soil, household items, and human clinical samples with accession numbers in GenBank (https://www.ncbi.nlm.nih.gov/nuccore; accessed on 8 December 2024) were obtained, and a phylogenetic tree was plotted. Evolutionary analyses were performed in MEGA11 [37].

### 2.3. Determination of Phenolic Compound Content of Muğla Propolis

#### 2.3.1. Propolis Sample Collection and Extraction

Propolis samples were collected in areas predominantly covered by red pine forests (*Pinus brutia*), as well as *Pistacia* spp. and *Liquidambar orientalis*, in heathland and scrubland, in the spring of Muğla/Akyaka, Türkiye. Propolis traps were placed on hives containing colonies of *Apis mellifera anatoliaca*, which is an ecotype from Muğla. The collected propolis was stored at +4 °C until extraction. The extraction method was performed as described by Sorucu and Oruç [38]. A grain mill was used to homogenize all collected brownish propolis samples. One hundred grams of propolis was weighed and added to 300 mL of 70% ethanol/water (*v*/*v*). The mixture was then shaken on a shaker (Nüve SL-35, Nüve A.Ş., Ankara, Türkiye) for 1 h, followed by ultrasonication (Bandelin Sonorex RK100, Bandelin electronic GmbH & Co. KG, Berlin, Germany) for 30 min. The extraction was filtered through filter papers (Whatman No. 1, UK) to remove wax and bee parts. To determine the resin ratio, the filtrate was poured into tared glass tubes and evaporated using a vacuum centrifuge (Jouan, RC 10-10). The extract, whose resin ratio was determined, was used in the antifungal activity study by making dose dilutions. The phenolic compounds were also determined in the propolis extract analyzed by Ultra Performance Liquid Chromatography-Mass Spectrometry (UPLC–MS/MS) (Eksigent–AB Sciex, Framingham, MA, USA).

#### 2.3.2. Chemicals and Reagents

The phenolic compounds, which were longifolene, beta caryophyllene oxide, kaempferol, t-cinnamic acid, galangin, apigenin, 3,4-dimethoxy cinnamic acid, t-p-coumaric acid, cape, luteolin, rutin, fisetin, rac-naringenin, quercetin, naringenin, genistein, formonetin, emodin, caffeic acid, 3-hydroxy-4-methoxy cinnamic acid, were purchased from Santa-Cruz Biotechnology. HPLC-grade methanol, ethanol, and formic acid were purchased from Merck (Darmstadt, Germany).

#### 2.3.3. UPLC-MS/MS Analyses and Method Validation

The extraction method was carried out according to the description provided by Sorucu et al. [39]. Chromatographic analysis was performed using the ekspert™ ultraLC with ekspert™ ultraLC 100 pump system, autosampler, and column oven (Eksigent-AB Sciex, Framingham, MA, USA). Chromatographic separation was performed on an Ultisil XB C18 column (2.1 × 50 mm, 5 μm). A linear gradient eluted the column. Mobile phase A (0.2% formic acid in water) and mobile phase B (0.2% formic acid in acetonitrile) were used at a 0.3 mL/min flow rate. The gradient elution was applied starting at 5% B (0–1 min), followed by 5–5% B (1–2 min), 5–95% B (2–5 min), 95–95% B (5–10 min), 95–5% B (10–10.1 min), and 5–5% B (10.1–12.1 min), and was stopped at 5–5% B (12.1–15 min). Analysis was carried out using a 3200 QTRAP mass spectrometer (AB Sciex, Foster City, CA, USA) equipped with an electrospray ionization (ESI) source. The mass spectrometer was operated in positive and negative ion modes, with a 5.5 kV potential applied to the electrospray ionization needle. One precursor ion (parent mass) and two daughter ions were monitored in multiple reaction monitoring (MRM) mode. The ionization source temperature was 500 °C. Nitrogen was used as curtain gas (30 psi), nebulizing gas (7 psi), collision gas (60 psi) and heating gas (60 psi).

### 2.4. Determination of In Vitro Inhibitory Effects of Muğla Propolis on Dermatophytes

The preparation of dermatophyte inocula was based on the protocol described by Aneke et al. [40], with minor modifications applied. Fourteen-day-old fresh cultures of the isolates were prepared on SDA medium. Sterile physiological saline (0.85% NaCl) was added onto the growing colonies, and the surface was gently scraped with the tip of a sterile Pasteur pipette. The resulting suspension was transferred into sterile tubes, and the mixture was allowed to stand for 15 min to enable the sedimentation of heavier hyphal fragments, allowing the conidia to remain in suspension. The supernatant was then filtered through Whatman No. 1 filter paper to remove coarse hyphal fragments. The filtrate obtained was adjusted to 2.4 McFarland, corresponding to approximately 1–5 × 10^6^ conidia/mL, using sterile physiological saline.

The in vitro inhibitory effects of the propolis extract against the dermatophyte isolates were evaluated using the agar dilution method through a mycelial growth inhibition assay, as described by Dudoit et al. [41]. For the test, 19 mL of sterilized SDA medium, cooled to below 50 °C, was supplemented with 1 mL of the propolis extract prepared at different dilutions (100, 50, 25, 12.5, and 6.25 mg/mL), and the plates were gently mixed by circular movements. Each Petri dish was inoculated with 10 µL of a vortexed fungal suspension (approximately 10^6^ conidia/mL), and the plates were incubated at 25 °C for 10 days. As a negative control, SDA medium containing 1 mL of 70% ethanol (without propolis extract) was used. At the end of the incubation period, the two perpendicular diameters (mm) of the fungal colonies were measured, and the mean values were recorded. The percentage of mycelial inhibition was calculated using the following formula:

$$Percentage\;inhibition=\frac{D_{0}-D_{propolis}}{D_{0}}\times 100$$

where *D*_0_ represents the colony diameter of the control, and *D_propolis_* represents the colony diameter of the treated samples. All experiments were performed in duplicate. Antifungal activity was evaluated descriptively based on the presence or absence of visible fungal growth and percentage mycelial growth inhibition. Inferential statistical analysis was not planned, as the experimental design was based on qualitative assessment of growth inhibition.

## 3. Results

### 3.1. Isolation Results and Morphological Characteristics of Dermatophyte Agents

**Figure 1:**
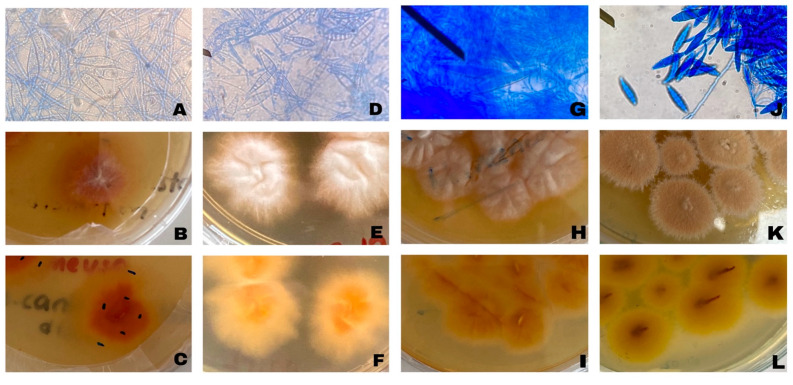
Representative microscopic and macroscopic morphological characteristics of dermatophyte field isolates obtained from cats. Microscopic images show hyphal and conidial structures used for routine morphological identification, while macroscopic images illustrate colony morphology on Sabouraud Dextrose Agar. These images are provided for morphological characterization. (**A**) MU1 microscopic image; (**B**) MU1 colony morphology front view; (**C**) MU1 colony morphology back view; (**D**) MI4 microscopic image; (**E**) MI4 colony morphology front view; (**F**) MI4 colony morphology back view; (**G**) MI7 microscopic image; (**H**) MI7 colony morphology front view; (**I**) MI7 colony morphology back view; (**J**) MU3 microscopic image; (**K**) MU3 colony morphology front view; (**L**) MU3 colony morphology back view.

A total of 30 skin scrapings were collected from cats and dogs. Among these animals, 22 were cats, and 8 were dogs. By the end of the incubation period, fungal growth was detected in 21 of these samples, beginning on the tenth day of incubation. However, no growth was observed in 9 samples after 4 weeks of incubation. Microscopic examination of preparations obtained from fungal cultures revealed that, based on macroconidial and hyphal morphology, three isolates (MU1, MI4, MI7) were identified as *Microsporum* spp., while one isolate (MU3) was classified as *Nannizzia* spp. The remaining fungal isolates were identified as non-dermatophytic fungi based on morphological examination and were therefore excluded from further analyses. All dermatophyte isolates characterized in this study were recorded as originating from cats. The macroscopic and microscopic appearances of the identified dermatophyte agents are presented in Figure 1.

### 3.2. Molecular Identification Results and Phylogeny of Dermatophyte Agents

The ITS DNA regions were amplified using ITS1 and ITS4 primers, resulting in fragments approximately 678 to 789 bp in length. A comparison of the obtained ITS nucleotide sequences with the NCBI GenBank database revealed that three of the isolates were identified as *M. canis*, while the fourth was identified as *N. gypsea*. The isolates were assigned the following GenBank accession numbers: PQ177464.1 (*M. canis* isolate MI7), PQ176821.1 (*M. canis* isolate MI4), PQ177471.1 (*M. canis* isolate MU1), and PQ177470.1 (*N. gypsea* isolate MU3). The isolation source, country of origin, and GenBank accession numbers for the species used in the phylogenetic tree analysis are provided in Table 1.

**Figure 2:**
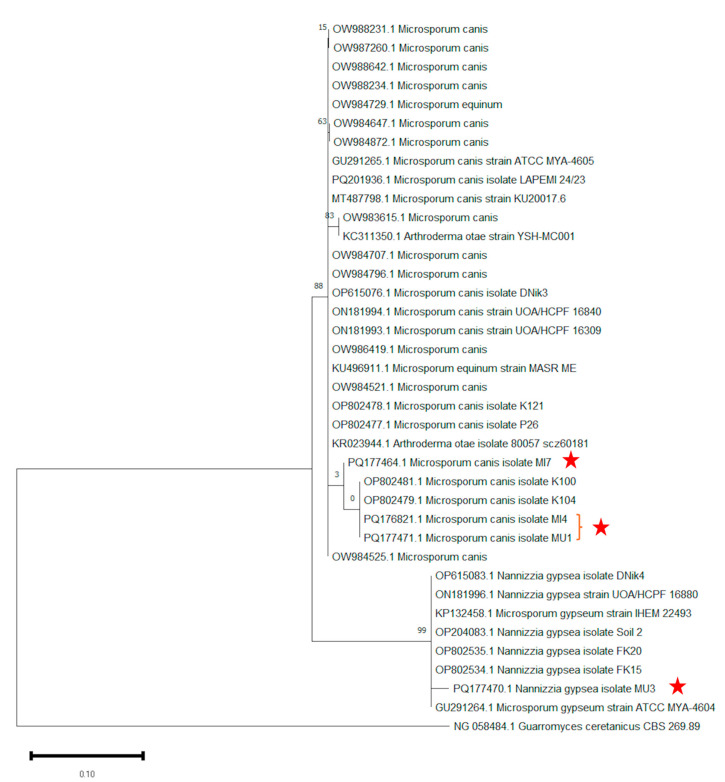
Phylogenetic tree showing the taxonomic positions of identified fungal species based on the ITS sequence. Outgroup *Guarromyces ceretanicus* CBS 269.89 was used as the outgroup. The evolutionary history was inferred by using the Maximum Likelihood method and Tamura–Nei model.

The evolutionary history was inferred by using the Maximum Likelihood method based on the Tamura–Nei model [42]. The tree with the highest log likelihood (−2670.65) is shown. The percentage of trees in which the associated taxa clustered together is shown above the branches. Initial tree(s) for the heuristic search were obtained automatically by applying Neighbor-Join and BioNJ algorithms to a matrix of pairwise distances estimated using the Tamura–Nei model, and then selecting the topology with the superior log-likelihood value. This analysis involved 38 ITS rDNA nucleotide sequences, including the sequences generated in the present study and reference sequences retrieved from GenBank. There were 986 positions in the final dataset. PQ177464.1 (*M. canis* isolate MI7), PQ176821.1 *(M. canis* isolate MI4), and PQ177471.1 (*M. canis* isolate MU1) were located in a separate branch from other *M. canis* strains in the tree, on the other hand, PQ177470.1 (*N. gypsea* isolate MU3) was located in a separate branch from other *N. gypsea* strains in the tree. *Guarromyces ceretanicus* CBS 269.89 was used as the outgroup in the phylogenetic tree drawing (Figure 2). Evolutionary analyses were conducted in MEGA11 [37].

### 3.3. Phenolic Compound Content Results of Muğla Propolis

**Figure 3:**
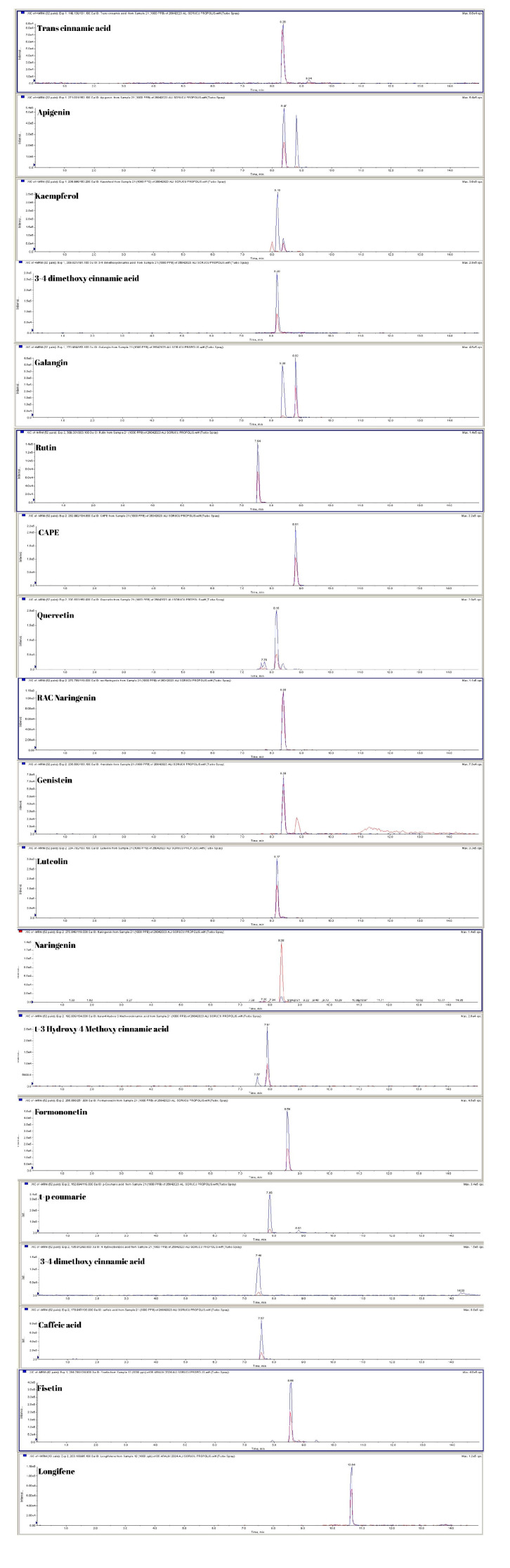
The spectra and ions of phenolic compounds were characterized, and the detection limits were established through serial dilutions of mixed phenolic compounds (MFC). The limits of detection (LOD) and quantitation (LOQ) were calculated using the 3 S/N (signal-to-noise ratio) and 10 S/N equations, respectively. The resulting LOD and LOQ values for phenolic compounds are summarized in Table 2. MFC was diluted with methanol to prepare calibration standards at concentrations of 250, 500, and 1000 ng/mL. The calibration curves for each phenolic compound were established through linear regression analysis (R2), with the R2 values detailed in Table 2 and Figure 3. Two types of propolis samples were randomly selected for recovery assessment, each spiked with MFC at three concentration levels (20, 10, and 5 μg/mL). Recovery rates of phenolic compounds ranged from 88% to 115%, and statistical comparisons between spiked and unspiked (blank) propolis samples were performed. The coefficient of variation (relative standard deviation, RSD) for the recovery data was less than 4.6%, while the RSD for retention time was below 1.8%. The repeatability of the method was evaluated by conducting multiple measurements of MFC, demonstrating satisfactory precision.

The phenolic compounds were characterized using UPLC-MS/MS analysis, revealing that the propolis sample exhibits a notably diverse phenolic profile. When classified by chemical group, phenolic acid derivatives were identified at especially high concentrations. Within this group, 3,4-dimethoxy cinnamic acid (772 µg/mL), caffeic acid (838 µg/mL), trans-cinnamic acid (406 µg/mL), and trans-p-coumaric acid (400 µg/mL) were identified as predominant compounds. Additionally, the phenolic compound caffeic acid phenethyl ester (CAPE) was present at a notably high concentration of 1050 µg/mL. Moreover, the phenolic acid derivative trans-3-hydroxy-4-methoxy cinnamic acid was detected at a concentration of 54 µg/mL. Flavonoids were identified as the predominant phenolic compound in the sample, with genistein (3540 µg/mL) being the most prevalent. Other flavonoids, such as kaempferol (84 µg/mL), galangin (91 µg/mL), apigenin (99 µg/mL), quercetin (150 µg/mL), luteolin (9 µg/mL), naringenin (9 µg/mL), and fisetin (10 µg/mL), were detected at varying concentrations. Among the flavonoid subclasses, formononetin (8 µg/mL), rutin (2 µg/mL), and rac-naringenin (1 µg/mL) were present at low but measurable levels. Among the terpenoid compounds identified, longifolene (114 µg/mL) and β-caryophyllene oxide (155 µg/mL) were particularly notable and detected in significant quantities within the propolis sample. Mass spectrometry parameters include collision energy (CE), collision cell exit potential (CXP), entrance potential (EP), declustering potential (DP), parent ion (Q1 Da), and daughter ion (Q3 Da), as detailed in Table 2. The spectrum and ion information for phenolic compounds are illustrated in Figure 3.

### 3.4. In Vitro Inhibitory Effects of Muğla Propolis on Dermatophytes

When the in vitro inhibitory effect of Muğla propolis on dermatophyte isolates was evaluated, complete growth inhibition (100%) was observed in all four field isolates at all tested concentrations (100, 50, 25, 12.5, and 6.25 mg/mL). In all propolis-treated plates, no visible colony growth was detected, and the mean colony diameter was recorded as 0 mm. In contrast, fungal growth was observed in the negative control plates, with mean colony diameters of 55 mm for the MU1 isolate, 64.75 mm for the MI4 isolate, 46.5 mm for the MI7 isolate, and 50 mm for the MU3 isolate. These findings confirm that the isolates retained normal growth capacity under the experimental conditions, while growth inhibition was observed following exposure to the propolis extract. No differences in susceptibility were observed among the tested dermatophyte isolates, and similar inhibitory outcomes were recorded across all concentrations evaluated.

## 4. Discussion

To determine whether the source of infection is zoophilic (animal-related), anthropophilic (human-related), or geophilic (soil-related), it is essential to identify dermatophytes at the species level accurately. This identification is crucial for implementing appropriate treatment and control precautions [43]. Because conventional laboratory methods for identifying dermatophytes can be slow and may lack specificity, molecular technologies, such as DNA sequencing techniques, are necessary for rapid and accurate identification of these fungi [44]. Amplification of the internal transcribed spacer region of nuclear ribosomal DNA (ITS rDNA) is considered the gold standard for dermatophyte identification [45]. Variations in the nucleotide composition of the ITS region have been effectively used to distinguish between species [46]. Qualitative (conventional) PCR methods for detecting and identifying dermatophytes typically target the ITS region, allowing for the identification of many isolates down to the species level. Primers designed to target the conserved regions of the ITS specific to dermatophytes facilitate the identification of dermatophyte-positive samples [47]. In the present study, the universal primer pairs ITS1 and ITS4 were used to amplify the specific marker and conserved sequence. Numerous studies have reported these primer pairs as widely used tools for fungal molecular diagnosis and identification [48]. Moreover, the extensive data available for this region in the GenBank database allows for meaningful comparisons of the sequences obtained [49]. In the present work, three *M. canis* isolates and one *N. gypsea* isolate were obtained from cats and confirmed molecularly. These findings aligned with previous reports indicating that cats were the principal reservoir for *M. canis*, while *N. gypsea*, although primarily soil-borne, was reported to cause opportunistic infections in cats [2,3,4,5,11,12,50].

Sequence analysis revealed a 99.73–100% similarity between the isolates in the present study and dermatophyte sequences recorded in GenBank from various countries, including Belgium, Greece, Thailand, New Zealand, Brazil, China, Luxembourg, Iran, Egypt, India, Germany, and Uganda. This high sequence similarity underscores the widespread distribution and genetic stability of species such as *M. canis* and *N. gypsea*, both of which are major etiological agents of ringworm in cats and dogs. Accordingly, infected cats pose a risk of transmission of dermatophytes to other animals and humans [51]. The phylogenetic clustering of the isolates with sequences from different hosts and geographical regions supports the high transmissibility of *M. canis*. As expected, the *N. gypsea* isolate clustered with soil-associated strains, consistent with its ecological niche [47]. Recent studies have also noted increasing rates of asymptomatic dermatophyte carriage in animals, which heightens the risk of zoonotic transmission to humans [52].

Antimicrobial resistance developing against antimicrobial agents in humans and animals has a significant impact on morbidity, mortality, and healthcare costs. In recent years, a notable increase in the incidence of fungal infections has been observed due to the rise in diseases that suppress the immune system or the widespread use of immunosuppressive treatments. Similarly, the widespread use of broad-spectrum antifungal compounds in veterinary practice has been associated with an increased incidence of antifungal resistance in fungal diseases observed in cats and dogs [53]. In the treatment of dermatophytosis in companion animals, antifungal drugs such as itraconazole, ketoconazole, and terbinafine are widely used [3,54]. In contrast, fluconazole is preferred as a first-line treatment in humans in many European countries [45,55]. Although azole resistance has been rarely reported, clinical evidence indicates that some *Microsporum canis* infections may be difficult to treat with azoles or terbinafine, and some antifungal resistance studies have reported that this agent exhibits low susceptibility to itraconazole or fluconazole. This situation has been interpreted as being associated with acquired azole resistance resulting from the repeated use of antifungal agents [40,45,56,57,58]. In parallel with this trend, research into alternative biological agents for the treatment of dermatomycotic infections, which are important from a public health perspective due to their zoonotic characteristics, has also increased significantly in recent years [30]. The antifungal potential of propolis against a range of dermatophytes, yeasts, and non-dermatophytic molds has been documented in several studies [27,28,31,59,60]. However, when compared with the substantial body of literature addressing the activity of propolis against *Candida* spp. and filamentous fungi, studies specifically examining its effects on dermatophytes particularly *M. canis* and *N. gypsea* remain relatively limited [27,28,29,30,32]. In Türkiye, the chemical composition of propolis samples from the Aegean region and their antifungal activity against *Fusarium oxysporum* have been characterized; although Muğla propolis demonstrated the highest antifungal activity in that study, no assessment against dermatophytes was undertaken [61]. However, in the present study, the antifungal activity of propolis was evaluated exclusively against molecularly confirmed dermatophyte field isolates, and no other fungal genera were included in the experimental design. To the best of our knowledge, the present study is the first to concurrently characterize the detailed phenolic and flavonoid profile of Muğla-origin propolis via UPLC-MS/MS and to evaluate its in vitro antifungal activity against field isolates of *M. canis* and *N. gypsea* obtained from cats.

The absence of a reference antifungal agent represents a limitation of the present study and restricts direct comparison of the inhibitory effect of propolis with conventional antifungal drugs. The inhibitory activity of Muğla propolis against dermatophyte isolates is suggested to be associated with the rich chemical composition of the extract, which includes phenolic acids, flavonoids, and terpenoids. UPLC–MS/MS analysis revealed particularly high concentrations of biologically active compounds, notably genistein and caffeic acid phenethyl ester (CAPE). Among the flavonoids identified, genistein was detected at the highest level. Previous research by Xiao et al. [62] demonstrated that genistein induces cell membrane damage and spore deformation in *T. mentagrophytes*. In addition, genistein has been reported as the predominant compound in Tunisian propolis, and propolis samples rich in phenolic compounds and flavonoids were shown to exhibit stronger antifungal activity against *Penicillium*, *Aspergillus*, and *Fusarium* species [63]. Taken together, these findings indicate that the high genistein content observed in the present study may play a contributory role in the inhibition of dermatophytes. CAPE is a polyphenolic ester and one of the principal constituents of propolis, which can also be synthesized under laboratory conditions. Its biological activities are largely attributed to the hydroxyl groups present on the catechol ring structure. CAPE has been reported to inhibit viability, biofilm formation, and cell survival in *Candida* species in a concentration and strain dependent manner [64]. In the present study, the detection of CAPE as the second most abundant compound suggests that it may represent one of the chemical factors contributing to the observed inhibitory effects against dermatophytes.

Other phenolic compounds also play an important role in explaining the antifungal activity. Although not focused on dermatophytes, Türk et al. [61] reported the presence of phenolic acids such as caffeic acid, coumaric acid, and cinnamic acid in propolis samples from the Aegean Region, in which varying degrees of antifungal activity were detected. Similarly, cinnamic acid derivatives, such as 3,4-dimethoxy cinnamic acid, identified in the present study, represent phenolic acids among the chemical constituents of Muğla propolis. Woźniak et al. [65] characterized various hydroxycinnamic acid derivatives, particularly coumaric acid, in propolis samples and demonstrated that phenolic compounds exert antifungal effects against different fungal species. Furthermore, Gargouri et al. [63] reported that propolis samples collected from various regions of Tunisia were rich in phenolic acids and flavonoids and exhibited significant antifungal properties. Taken together, although these phenolic acids were not directly tested against dermatophytes, their high levels detected in the present study suggest that, due to their structural characteristics, they may contribute to the observed antifungal activity.

An examination of the flavonoid composition of Muğla propolis revealed that compounds such as galangin, kaempferol, apigenin, rutin, luteolin, quercetin, and naringenin were present at considerable levels. In line with these findings, Woźniak et al. [65] reported that several flavonoids, including apigenin, chrysin, galangin, kaempferol, and pinocembrin, showed a statistically significant association with antifungal activity based on multivariate analysis of variance (MANOVA). Galangin and pinocembrin were also identified in Argentine propolis by Agüero et al. [29], who reported that these propolis samples exerted inhibitory effects against *M. gypseum*, *T. tonsurans*, and *T. mentagrophytes*. In addition, Bergamini et al. [32] detected compounds such as apigenin, galangin, kaempferol, luteolin, rutin, and quercetin diglucoside in propolis collected during different seasons in Brazil and reported that these propolis samples exhibited fungicidal activity against *T. rubrum*. These findings indicate that the flavonoid diversity identified in the present study is consistent with the observed inhibition of dermatophytes. Notably, a dermatophyte-specific study focusing on apigenin is also available. Singh et al. [66] reported that topical application of apigenin isolated from the plant *Terminalia chebula* resulted in complete recovery within 12–16 days in mice infected with *T. mentagrophytes*. The presence of apigenin in Muğla propolis is therefore noteworthy, given the well-documented antifungal activity of this compound against dermatophytes. Among the terpenoids, longifolene and β-caryophyllene oxide are also of particular interest. Longifolene was identified by Bintang et al. [67] in a propolis mixture prepared with various plant sources, and the mixture was reported to exhibit antifungal activity against *Candida albicans*. Formononetin, on the other hand, was identified in Brazilian red propolis by Neves et al. [68] and was reported to display antifungal activity against *Candida* species. Furthermore, formononetin was isolated from the acetate fraction, and its antifungal activity was independently evaluated, confirming its antifungal efficacy.

## 5. Conclusions

When all findings are considered together, the ITS-based phylogeny revealed that the isolates in this study exhibit a high level of genetic similarity, both intra-group and with dermatophyte strains documented from various countries. The antifungal activity observed against molecularly confirmed dermatophyte field isolates suggests a potential relevance in the context of increasing antifungal resistance. However, since reference strains were not included and individual propolis components were not tested separately, the present study does not provide experimental evidence for synergistic antifungal interactions. In light of the growing global problem of antimicrobial resistance and considering the therapeutic potential of Muğla propolis in the treatment of dermatophyte infections, more comprehensive studies using isolated compounds are needed to better elucidate its antifungal mechanisms.

## 6. Study Limitations

The present study has certain methodological limitations that should be acknowledged. First, a standard reference antifungal agent was not included in the agar dilution assay; therefore, direct quantitative comparison between the inhibitory effect of Muğla propolis and conventional antifungal drugs could not be performed. In addition, minimum inhibitory concentration (MIC) values were not determined using standardized broth microdilution methods (e.g., CLSI or EUCAST formats), as the experimental design was intended as an initial agar-based screening of antifungal activity rather than a formal susceptibility assessment. In addition, reference dermatophyte strains were not included, and individual propolis constituents were not tested separately. Consequently, potential synergistic or additive antifungal interactions among the bioactive compounds identified in the extract could not be experimentally verified. Therefore, the findings of this study should be interpreted within the context of an exploratory in vitro evaluation. Despite these limitations, the results provide preliminary evidence supporting the antifungal activity of region-specific Muğla propolis against molecularly confirmed dermatophyte field isolates and may serve as a foundation for future studies incorporating standard antifungal controls, reference strains, MIC determination, and isolated propolis constituents.

## Figures and Tables

**Table 1:** The isolation source, country of origin, partial sequence size, and GenBank accession numbers for the species used in the phylogenetic tree analysis.

Species	Isolation Source	Country	Partial Sequence (bp)	GenBank Accession Number
*Microsporum canis* isolate MI7	cat	Türkiye (this study)	743	PQ177464.1
*Microsporum canis* isolate MI4	cat	Türkiye (this study)	789	PQ176821.1
*Microsporum canis* isolate MU1	cat	Türkiye (this study)	742	PQ177471.1
*Microsporum canis* isolate DNik3	guinea pig/hamster	Iran	745	OP615076.1
*Microsporum canis*	dwelling environment, couch	Belgium	751	OW986419.1
*Microsporum canis*	Persian cat	Belgium	762	OW984525.1
*Microsporum canis* strain UOA/HCPF 16840	homo sapiens/bronchial secretions	Greece	761	ON181994.1
*Microsporum canis* strain UOA/HCPF 16309	homo sapiens/foot trauma	Greece	761	ON181993.1
*Microsporum canis*	human hair	Belgium	736	OW988642.1
*Microsporum canis*	human skin	Belgium	734	OW983615.1
*Microsporum canis* strain KU20017.6	cat/pus and grains: pseudomycetoma	Thailand	726	MT487798.1
*Microsporum canis*	human thorax	Belgium	738	OW988231.1
*Microsporum canis*	human skin (arm	Belgium	736	OW988234.1
*Microsporum canis*	guinea pig (cavia porcellus)	Belgium	744	OW984647.1
*Microsporum equinum*	horse cutaneous lesion	Belgium	744	OW984729.1
*Microsporum canis*	human scalp	New Zealand	741	OW984796.1
*Microsporum canis*	dog microspory	Belgium	742	OW984872.1
*Microsporum canis* isolate LAPEMI 24/23	canine lesion	Brazil	736	PQ201936.1
*Microsporum canis*	rabbit hair	Belgium	745	OW987260.1
*Microsporum canis* strain YSH-MC001	homo sapiens/scalp of daughter	China	736	KC311350.1
*Microsporum canis*	human foot	Luxembourg	755	OW984707.1
*Microsporum canis*	cat (felis catus)	Belgium	750	OW984521.1
*Microsporum canis* isolate P26	cow	Iran	738	OP802477.1
*Microsporum canis* isolate K121	dog	Iran	738	OP802478.1
*Microsporum canis* isolate 80057 scz60181	homo sapiens/hair	China	738	KR023944.1
*Microsporum equinum* strain MASR ME	equine/horse ringworm lesion	Egypt	750	KU496911.1
*Microsporum canis* isolate K104	dog	Iran	737	OP802479.1
*Microsporum canis* isolate K100	cat	Iran	737	OP802481.1
*Microsporum canis strain ATCC* MYA-4605	head	Germany	721	GU291265.1
*Nannizzia gypsea* isolate MU3	cat	Türkiye (this study)	678	PQ177470.1
*Nannizzia gypsea* strain UOA7HCPF 16880	homo sapiens/skin scales	Greece	690	ON181996.1
*Nannizzia gypsea* isolate DNik4	rodents	Iran	686	OP615083.1
*Nannizzia gypsea* isolate FK15	dog	Iran	666	OP802534.1
*Nannizzia gypsea* isolate FK20	horse	Iran	666	OP802535.1
*Nannizzia gypsea* isolate Soil 2	soil	India	664	OP204083.1
*Microsporum gypseum strain ATCC MYA-4604*	skin	Uganda	650	GU291264.1
*Guarromyces ceretanicus* CBS 269.89 (as outgroup)	soil	Chile	809	NG_058484.1

**Table 2:** The method, MS/MS parameters and results of phenolic compounds.

Phenolic Compounds	LOD ng/mL	R^2^	Ion Mode	Q1 (Da)	Q3 (Da)	DP (V)	EP (V)	CEP (V)	CE (V)	CXP (V)	Results (µg/mL)
	**LOQ ng/mL**										
Longifolene	7.16	0.9978	P	203.19	95.1	51	8	14	19	4	114
	21.48				105	51	8	14	21	4	
β-Caryophyllene oxide	4.18	1	P	221.18	203.1	26	9	14	13	8	155
	12.54				105.1	26	9	14	33	4	
Kaempferol	13.45	0.9980	P	287	153.2	86	5.5	14	41	4	84
	40.35				121.2	86	5.5	14	47	4	
t-cinnamic acid	8.4	0.9957	P	149.11	131.1	31	8	12	13	4	406
	25.2				102.9	31	8	12	27	4	
Galangin	7.6	0.9992	P	270.98	153.1	86	11.5	20.6	41	4	91
	22.8				77	86	11.5	20.6	73	4	
Apigenin	2.3	0.9991	P	271	153.1	71	7	20.6	45	4	99
	6.9				119	71	7	20.6	43	4	
3–4 dimethoxy cinnamic acid	16.3	0.999	P	209.02	191.1	36	5.5	18.8	13	4	772
	48.9				163.2	36	5.5	18.8	25	4	
*t*-p coumaric	4.36	1	N	162.79	118.9	−35	−2	−14	−20	0	400
	13.08				93	−35	−2	−18.2	−38	0	
CAPE	14.9	0.9995	N	282.88	135	−50	−8.5	−16	−34	0	1050
	44.7				179	−50	−8.5	−16	−20	0	
Luteolin	0.65	0.9997	N	284.78	133.1	−85	−11	−16	−44	0	9
	1.95				151	−85	−11	−16	−34	0	
Rutin	1.13	0.999	N	609.03	300.1	−105	−8	−22	−54	−2	2
	3.39				271.1	−105	−8	−22	−78	−2	
Fisetin	4.1	1	N	284.79	134.8	−95	−11	−16	−30	0	10
	12.3				121.1	−95	−11	−16	−38	0	
RAC Naringenin	0.43	0.999	N	270.79	119	−70	−10	−16	−58	0	1
	1.29				150.8	−70	−10	−16	−14	0	
Quercetin	7.98	0.9999	N	300.83	150.9	−65	−9.5	−16	−30	0	150
	23.94				179.1	−65	−9.5	−16	−22	0	
Naringenin	1.67	0.9999	N	270.85	151	−65	−11	−14	−26	0	9
	5.01				119	−65	−11	−14	−34	0	
t-3 Hydroxy 4 Methoxy cinnamic acid	3.5	0.9996	N	192.84	134	−50	−4.5	−12	−24	−2	54
	10.5				117	−50	−4.5	−12	−24	0	
Caffeic acid	10.78	0.9996	N	178.95	135	−35	−4.5	−12	−22	0	838
	32.34				134.1	−35	−4.5	−12	−30	0	
Formononetin	3.12	0.9965	N	266.89	251.9	−65	−9.5	−14	−16	−2	8
	9.36				222.9	−65	−9.5	−14	−36	−2	
Genistein	14.8	0.9934	N	268.8	133.1	−70	−11	−14	−42	0	3540
	44.4				63.1	−70	−11	−14	−52	0	

LOD: Limit of detection, LOQ: limit of quantitation, Q1 (Da): Parent ion Q3 (Da): daughter ions, DP (volt): Declustering potential, EP (volt): Entrance Potential CEP (volt) CE (volt): Collision energy, CXP (volt): Collision Cell Exit Potential, P: positive ion mode, N: Negative ion mode.

## Data Availability

The original contributions presented in this study are included in this article. Further inquiries can be directed to the corresponding author.
